# Supplementary material for: Content-rich biological network constructed by mining PubMed abstracts
Source: BMC Bioinformatics. 2004 Oct 8;5:147. doi: 10.1186/1471-2105-5-147 (PMC528731; doi:10.1186/1471-2105-5-147)
Supplement: Additional File 2 — The original results of the above study (non-essential files are deleted to keep the file size under the limit set by BMC bioinformatics). [file 1471-2105-5-147-S2.bz2 › chilibotAdditionalFile2/dip05/176ID8876196E602/html/RAF1_NRAS.html]

 


 **RAF1** and **NRAS** 
  
Found 7 abstracts in PubMed, retrieved 05.  
 

 What does Google say? 
 PDF only 
| .edu only 

---

**Interactive relationship** (e.g. stimulation, inhibition, etc)

**Inhibitory relationship**- Furthermore, we identified common and distinct targets in cells transformed by mutant HRAS, KRAS and  **NRAS** , as well as 61 putative target genes controlled by the RAF  [ **RAF1** ]  MEK ERK pathway in reverted cells treated with the MEK specific inhibitor PD 98059.  Ref: 10655059 Nat Genet, 2000
**Neutral relationship**- As for other genes, amplification was detected for int 2 also known as WNT2 seven 14% of 50, erbB 2 also known as ERBB2 three 6% of 50, N ras also known as  **NRAS**  one 2% of 50, L myc also known as MYCL1 one 2% of 50, and raf 1  [ **RAF1** ]  also known as  **RAF1**  one 2% of 50.  Ref: 8064891 J Natl Cancer Inst, 1994

**Non-interactive relationship** (e.g. studied together, co-existance, homology, etc.)

- This included oncogene copy number gains of FGFR1 8p11 in three cases, and  **NRAS**  1p13.2, MYCN 2p24.1,  **RAF1**  3p25, CTSB 8p22, FES 15q26.1, and CBFA2 21q22.3 in two cases.  Ref: 12696066 Genes Chromosomes Cancer, 2003
- Other high level amplified oncogenes included  **NRAS** ,  **RAF1** , MYB, EGFR, FGF4, EMS1, and D17S167.  Ref: 11836556 Int J Oncol, 2002
- DNA probes for the  **NRAS** , HRAS, KRAS2, LCK,  **RAF1** , MET, MYCL1, MYCN, MYB, ERBB2, FOS, CSF1R, and SRC protooncogene loci.  Ref: 8095010 Cytogenet Cell Genet, 1993
